# Diverse potential of secretome from natural killer cells and monocyte-derived macrophages in activating stellate cells

**DOI:** 10.3389/fimmu.2024.1232070

**Published:** 2024-04-03

**Authors:** Julia Sauer, Agnes A. Steixner-Kumar, Svenja Gabler, Maciej Motyka, Jörg F. Rippmann, Stefan Brosa, Dennis Boettner, Tanja Schönberger, Charlotte Lempp, Vanessa Frodermann, Eric Simon, Oliver Krenkel, Ehsan Bahrami

**Affiliations:** ^1^ Boehringer-Ingelheim Pharma GmbH & Co. KG, Biberach, Germany; ^2^ Ardigen S.A., Cracow, Poland

**Keywords:** NASH - non-alcoholic steatohepatitis, inflammation, monocyte-derived macrophage (MDM), NK cells, liver fibrosis

## Abstract

Chronic liver diseases, such as non-alcoholic steatohepatitis (NASH)-induced cirrhosis, are characterized by an increasing accumulation of stressed, damaged, or dying hepatocytes. Hepatocyte damage triggers the activation of resident immune cells, such as Kupffer cells (KC), as well as the recruitment of immune cells from the circulation toward areas of inflammation. After infiltration, monocytes differentiate into monocyte-derived macrophages (MoMF) which are functionally distinct from resident KC. We herein aim to compare the *in vitro* signatures of polarized macrophages and activated hepatic stellate cells (HSC) with *ex vivo*-derived disease signatures from human NASH. Furthermore, to shed more light on HSC activation and liver fibrosis progression, we investigate the effects of the secretome from primary human monocytes, macrophages, and NK cells on HSC activation. Interleukin (IL)-4 and IL-13 treatment induced transforming growth factor beta 1 (TGF-β1) secretion by macrophages. However, the supernatant transfer did not induce HSC activation. Interestingly, PMA-activated macrophages showed strong induction of the fibrosis response genes *COL10A1* and *CTGF*, while the supernatant of IL-4/IL-13-treated monocytes induced the upregulation of *COL3A1* in HSC. The supernatant of PMA-activated NK cells had the strongest effect on *COL10A1* induction in HSC, while IL-15-stimulated NK cells reduced the expression of *COL1A1* and *CTGF*. These data indicate that other factors, aside from the well-known cytokines and chemokines, might potentially be stronger contributors to the activation of HSCs and induction of a fibrotic response, indicating a more diverse and complex role of monocytes, macrophages, and NK cells in liver fibrosis progression.

## Graphical Abstract

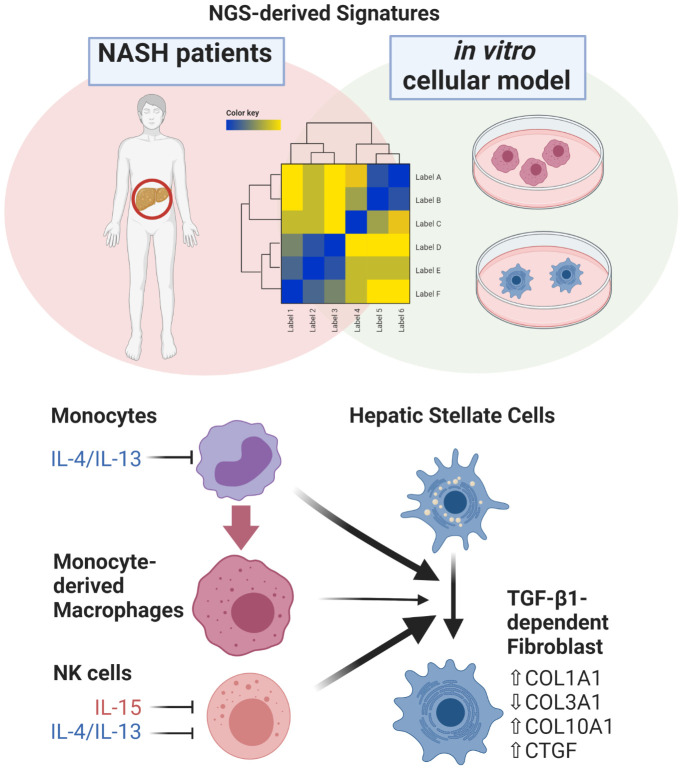

## Highlights

- A comprehensive analysis of RNA-Seq-derived gene signatures of *in vitro*-activated primary human monocytes, macrophages, hepatic stellate cells, and human NASH liver biopsies provides novel insights into the role of immune cell-mediated fibrosis progression.
- Pairwise gene-cluster enrichment analysis indicates a continuous loss of IL-4/IL-13 polarized monocyte-derived macrophage signature during fibrosis progression in human NASH liver biopsies.
- In comparison to monocytes and monocyte-derived macrophages, NK cells display more prominent potential in activating primary human hepatic stellate cells *in vitro*.
- Monocyte-derived macrophages and NK cells overactivated by PMA induce a strong fibrotic response in primary human HSC, while IL-4/IL-13-polarized cells lack this ability.

## Introduction

The recruitment of inflammatory immune cells from the circulation to the liver is considered as one of the hallmark characteristics of chronic fibrotic liver diseases such as non-alcoholic steatohepatitis (NASH) or viral hepatitis (1–3). Following the initial hepatocyte damage, local immune cells become activated and secrete cytokines and chemokines, which then result in the recruitment of immune cells including T cells, monocytes, and natural killer (NK) cells from the circulation toward areas of inflammation (4). Macrophages have been considered as key players in liver fibrosis progression, controlling the activation of hepatic stellate cells (HSC) and other liver fibroblasts, which, in turn, leads to increased collagen deposition and extracellular matrix buildup (5). Transforming growth factor beta (TGF-β1) plays a key role in activating HSCs via the TGFR-SMAD pathways, which ultimately leads to an upregulation of collagens, α-SMA, or CTGF upon activation (6, 7).

Besides the well-described pro-fibrotic properties of monocyte-derived macrophages (MoMF), recent data indicate a protective and restorative role of macrophages during liver fibrosis (8–10). The pool of liver macrophages during hepatic fibrosis consists of infiltrating MoMF as well as yolk-sac-derived tissue-resident Kupffer cells (KC) (11). After infiltration, MoMF display high plasticity and an adaptive inflammatory phenotype. Depending on the local microenvironment (3), MoMF undergo phenotypic maturation toward a restorative pro-resolution phenotype over time, characterized by a dampened response to inflammatory stimuli, reduced fibrogenic potential, and enhanced efferocytosis capacity (12). Besides MoMF, NK cells also play a role in regulating liver fibrosis via their cytotoxic killing of hepatic stellate cells (HSCs) and cross-talk with other hepatic cells (13, 14).

A growing body of evidence highlights the emergence of triggering receptors expressed on myeloid cells 2 (TREM2)^+^ lipid-associated macrophages (LAM) during the course of metabolic-associated liver disease including NASH. These macrophages are characterized by limited inflammatory and potentially protective capacity compared to other macrophage subsets (15, 16). During chronic liver diseases, the constant influx of monocytes toward areas of inflammation leads to an imbalance between resident KC and MoMF (17). Controversially, scar-associated TREM2^+^ macrophages with an increased fibrotic potential have also been described (18).

Transcriptome analysis by next-generation sequencing (RNA-Seq) has been established as the state-of-the-art genomics assay to quantify gene expression at the bulk sample level or even at single-cell resolution. Accordingly, we have applied RNA-Seq to systematically analyze and compare gene signatures observed in human NASH livers *versus* gene signatures observed *in vitro* in cell culture models. To shed some light on the functional role of MoMF in the pathophysiology of liver inflammation and NASH, we analyzed the enrichment of *in vitro*-derived signatures from polarized MoMF, resting HSC, and activated fibroblasts in human NASH liver biopsies across the full spectrum of fibrosis. Furthermore, we validated our findings by assessing the potential of monocytes, MoMF, and NK cells to induce HSC to fibroblast transformation *in vitro*.

We found that human NASH signatures significantly overlap with TGF-β1 response genes in HSC. Moreover, human NASH liver cells are characterized by a lack of genes highly expressed in macrophage colony-stimulating factor 1 (M-CSF)-, interleukin (IL)-4-, and IL-13-stimulated MoMF in contrast to the gene signatures of monocytes, which are highly enriched, indicating a lack of tissue-repair macrophages during NASH progression. In addition, the functional assessment of the fibrotic potential of monocytes, MoMF, and NK cells showed that the secretion of TGF-β1 does not correlate with their capacity to induce HSC activation. Furthermore, IL-4/IL-13-stimulated MoMF do not induce HSC activation *in vitro*. In conclusion, our data indicate that IL-4/IL-13-stimulated MoMF have restorative functions, but their quantity could be insufficient to cope with the worsening of chronic inflammation in late-stage NASH.

## Materials and methods

### Primary cell culture

Human monocytes were obtained either from purchased frozen peripheral blood mononuclear cells (PBMCs; Lonza, #CC-2702) or directly from fresh blood from healthy human donors. The blood donation and further handling were approved by the blood donation service from Boehringer Ingelheim Pharma GmbH and Co. KG in regard to local regulations and following ethical standards. CD14+ monocytes were isolated using the Human Pan Monocyte Isolation MACS Kit (Miltenyi Biotec, #130-096-537). The isolation of CD56+ NK cells was performed by using two kits from Miltenyi: Human CD56 Microbeads and Human NK Cell Isolation Kit (Miltenyi Biotec, #130-050-401 and #130-092-657), according to the manufacturer’s protocol, and the cells were immediately used for further experiments. The monocytes were cultured in X-Vivo™ 15 media (Lonza, #BE02-060F) containing 1% PenStrep (Gibco, #15140-122) and 2% HSA (Gibco, #H5422). For differentiation into macrophages, 100 ng/mL rhM-CSF (macrophage colony-stimulating factor) (R&D Systems, #216-MC) was added for 7 days (19). NK cells were cultured in RPMI 1640 medium (Lonza, #BE12-702F) containing 10% FCS (Biological Industries, #04-0001-1A) and 1% PenStrep.

Primary human HSCs, purchased from Zenbio (Zenbio, #HP-F-S), were used for the supernatant transfer assay between passages 2 and 5. The cells were cultured in SteCM Stellate Cell Medium (ScienCell™, #5301) containing 2% FBS (ScienCell™, #0010), 1% P/S Solution (ScienCell™, #0503), and 1% SteCGS (ScienCell™).

### RNA isolation and analysis

To extract RNA, cells were harvested, washed with phosphate-buffered saline (PBS), and lysed in RLT Plus Buffer (Qiagen, #1053393), followed by extraction of RNA using the RNeasy^®^ Mini Kit (Qiagen, #74106) according to the manufacturers’ protocol. The extracted RNA samples were quantified by using NanoDrop™ 8000 Spectrophotometer (Thermo Fisher Scientific) or NanoPhotometer^®^ N120 (Implen). For gene expression analysis, complementary DNA (cDNA) was synthesized using the High-Capacity cDNA Reverse Transcription Kit (Applied Biosystems™, #4368813) followed by qPCR (quantitative reverse transcription polymerase chain reaction) using TaqMan^®^ real-time PCR assay (Thermo Fisher, #4331182) for the following genes: COL1A1, COL3A1, COL10A1, ACTA2, and CTGF. The ΔΔCt method was used to calculate the relative expression levels using glyceraldehyde-3-phosphate dehydrogenase as the housekeeping gene. In brief, the Ct values were normalized to the housekeeping control gene GAPDH (delta CT value). The fold change was calculated with the 2-ΔCt/ΔCt equation, which means that each delta Ct value was normalized to an untreated control (medium control).

### RNA sequencing analysis of *in vitro*-stimulated HSC and macrophages

For sequencing library preparation, 100 ng of total RNA was used as input for the TrueSeq RNA Sample Prep Kit v2-Set B (RS-122-2002, Illumina Inc., San Diego, CA, USA), producing a 275-bp fragment including adapters in average size. Eight libraries were normalized and pooled together on a single lane and sequenced as 52-bp single reads and seven bases index read on an Illumina HiSeq2000 instrument using the TruSeq SBS Kit HS- v3 (50-cycle) (FC-401-3002, Illumina Inc., San Diego, CA, USA).

The human NASH data set was previously published (20) and downloaded from GEO under accession number GSE135251.

### RNA-Seq data analysis

A detailed description of the RNA-Seq analysis pipeline has been previously published (21). Briefly, reads passing the quality control filter were mapped against GRCh38 (human data). The gene expression levels were quantified based on genome annotation files from Ensembl version 86. For hierarchical clustering of the HSC and macrophage data, all genes with a significant differential expression (adjusted *p*-value ≤ 0.05, |log_2_foldchange| ≥0.5) in at least one of the conditions (treated vs. control) were selected. For the human NASH data set (20), we included all genes differentially expressed in at least one condition of F1–F4 vs. F0 (adjusted *p*-value ≤ 0.05, |log2FoldChange| ≥0.2).

Clustering was performed using DEGreport2 (v1.30.0) as described in (22). In all cases, we kept only clusters with more than 20 genes and made a pairwise comparison of MP and HSC clusters against NASH clusters by applying hypergeometric testing. We used EnhancedVolcano (23) (v1.12.0) to plot the differential expression results and ComplexHeatmap (24) (v2.10.0) to create heat maps.

We annotated all clusters by functional pathways from Reactome (25) database using clusterProfiler (26) (v4.2.2) and utilized enrichplot (27) (v1.14.1) to group similar pathways. Pairwise enrichment of human NASH clusters in gene clusters from the primary cell assays has been calculated using hypergeometric testing with R/Bioconductor.

### Cell analysis by flow cytometry

Immune cell populations after magnetic-activated cell sorting (MACS) isolation were determined. MA900 Multi-Application Cell Sorter (Sony Biotechnology) was used to detect the differently stained cells. For each sample, a stained and an unstained control were analyzed. The cells were transferred to U-bottomed flow cytometry (FACS) tubes and were incubated with human FcR Block (Miltenyi Biotec, #130-059-901) for 10 min at 4°C. Viability stain took place for 30 min at 4°C using LIVE/DEAD™ Fixable Yellow Dead Cell Stain Kit (Invitrogen, #L34959). Staining was performed as follows: 5 µL of CD19 Antibody (BD Horizon™ BB515 Mouse Anti-human CD19, #564456), 20 µL of CD3 Antibody (BD Pharmingen™ PE Mouse Anti-Human CD3, #555340), 5 µL of CD56 Antibody (BD Pharmingen™ PE-Cy™ 7 Mouse Anti-Human CD56, #557747), 5 µL of CD14 Antibody (BD Horizon™ BV421 Mouse Anti-Human CD14, #565283), 5 µL of CD45 Antibody (BD Horizon™ BV786 Mouse Anti-Human CD45, #563716) in 110 µL FACS buffer [PBS + 0.5% FCS (Biological Industries) + 2 mM EDTA (Sigma-Aldrich, #E7889)] per sample. After 15–20 min at 4°C, the samples were washed, centrifuged, and suspended for measurement in FACS buffer. For each sample, 100,000 events were measured. Data were analyzed using the FlowJo_v10.7.1 software (FlowJo, LCC).

### Immune cell activation

Macrophages were seeded either 1 day before stimulation or directly before stimulation into 96-well plates. Stimulation was done in 96-well plates for 6 h, and the medium was changed and further cultivated for additional 18 h. Immune cells were stimulated with rhIL-4 (2,000 U/mL, R&D Systems, #204-IL), rhIL-13 (200 U/mL, R&D Systems, #213-ILB), rhTNF-α (1,600 U/mL, R&D Systems, #210-TA), PGE_2_ (2 µg/mL, Tocris, #2296), phorbol 12-myristate 13-acetate (PMA, 64 ng/mL, Sigma Aldrich, #P8139), rhIL-15 (200 U/mL, R&D Systems, #247-IL), or rhIFN-γ (200 U/mL, R&D Systems, #285-IF).

### Cytokine analysis

To quantitate the secreted components of differently activated immune cells, a Meso Scale Discovery multiplex cytokine immunoassay was used. For these measurements, the U-PLEX^®^ Custom Biomaker (hu) Assay, Sector #K1067M-4 (with Spots IL-1β, IL-6, TNF-α + five open spots) (Meso Scale Discovery), was used following the manufacturer’s instructions. Data were analyzed using Excel (Microsoft Office) and Xlfit ^®^ (IDBS) add-in with the Cubic Model. Open spots were filled with human IL-10: U-PLEX^®^ Human IL-10 Antibody Set (Meso Scale Discovery, #B21TZ-3), human IL-8: U-PLEX^®^ IL-8 Human Antibody Set (Meso Scale Discovery, #B21TY-3), human granzyme A: U-PLEX^®^ Human Granzyme A Antibody Set and R-PLEX^®^ Human Granzyme A Antibody Set (Meso Scale Discovery, #B22G8-3), human IL-15: U-PLEX^®^ Human IL-15 Antibody Set (Meso Scale Discovery, #B21UR-3), and human IFN-γ: U-PLEX^®^ Human IFN-γ Antibody Set (Meso Scale Discovery, #B21TT-3).

### Supernatant transfer experiment

Primary HSCs were seeded in 24-well plates at a density of 60,000 cells per well. After 2 h, the medium was replaced for starvation (w/o FBS, SteCGS) for an additional 22 h. The supernatant of stimulated immune cells was added in 1:6 dilution and incubated for 48 h. As positive control rhTGF-β1 was used at the indicated concentrations. 

### BrdU incorporation assay

BrdU incorporation assay for analysis of proliferation (Roche, #11647229001; Roche) was performed according to the manufacturer’s guidelines with an incorporation time of 16 h and a total stimulation time with the conditioned medium of 24 h. Four independent HSCs were used for the assay.

### Scratch (cell migration) assay

Approximately 30,000 HSCs (three donors) were seeded on a special 96-well plate (nr) and starved in 0.1 FCS media overnight. To set the scratch, a 96-pin wound-making tool (Incucyte^®^ WoundMaker, Essen bioscience) was used, and the cells were monitored by life cell imaging over 48 h in IncuCyte S3 system. Analysis of relative wound density over time was conducted by using the Integrated Cell Migration analysis module (Satorius, catalogue no. 9600-0012).

### Animals

Female C57BL/6J mice (8–13 weeks) were obtained from Charles River (Sulzfeld, Germany or US). Groups of two to five mice were housed under specific pathogen-free conditions in individually isolated ventilated cages with chow and unrestricted access to water *ad libitum*. The animals were kept under constant temperature (22°C), humidity (55%), and light conditions (12-h day/night cycle). After an acclimatization period of at least 4 days after their arrival in the facility, the animals were used for the experiments. Cervical dislocation euthanasia of the mice was performed [euthanized under isoflurane-O2 (CP-Pharma, Burgdorf, Germany; Nicholas Piramal, London, UK) anesthesia]. After sacrifice, the livers were collected in cold University of Wisconsin (UW) preservation solution (DuPont Critical Care, Waukegab, IL, USA) and kept on ice until precision-cut liver slices were prepared. All experiments were approved by the animal welfare officers in Boehringer Ingelheim Pharma GmbH und Co KG as well as the Animal Review Committee of the German government and were performed according to the German Animal Protection Law.

### Preparation of precision-cut liver slices

Precision-cut liver slices (PCLS) were prepared from the whole liver with a Krumdieck tissue slicer (Alabama Research and Development). The PCLS had the following characteristics: diameter, 5 mm; thickness, 250–300 um; weight, 4 to 5 mg. The PCLS were incubated individually in 12-well plates in 1.3 mL of Williams Medium E (Gibco, #12551-032) supplemented with 10% Special HI fetal calf serum (FCS; Gibco, #16140-071), 25 mM D+glucose (Sigma, #68769-100ML), 2 mM GlutaMAX™ (Gibco, #31966-021), and 50 µg/mL gentamycin (Invitrogen). The PCLS were exposed to 100 ng/mL rmM-CSF (R&D Systems, #416-ML/CF), 118 ng/mL rmIL-4 (R&D Systems, #404-ML-010/CF), 256 ng/mL rmIL-13 (R&D Systems, #413-ML-005/CF), 20 ng/mL rmTNFα (R&D Systems, #410-MT/CF), and 100 ng/mL PMA (Sigma, #P8139). The PCLS were cultured for the indicated times in an incubator (Binder, Tuttlingen, Germany) at 37°C, with 90% O_2_ and 5% CO_2_, and horizontally shaken at 60 rpm.

The viability of PCLS was assessed by quantification of adenosine triphosphate (ATP) using a bioluminescence kit (Roche #11699695001). The obtained ATP value (pmol) was normalized to the total protein content (μg, measured with the Lowry method).

### Analysis of mRNA expression using qRT-PCR

Quantitative reverse transcription polymerase chain reaction (qPCR) was performed in order to assess the gene expression of several markers related to fibrosis, inflammation, and fat metabolism in *in vitro*-generated macrophages and PCLS. QRT-PCR is a quantitative method based on the amplification reaction of gene sequences and the increase in fluorescence signal.

Regarding RNA isolation from PCLS, three PCLS were pooled, lysed with 450 µL RLT buffer with 1% β mercaptoethanol, and snap-frozen. Using the RNeasy Mini Kit (Qiagen, #74104), RNA was isolated. RNA quantity and quality (A260/280) were determined via Nanodrop.

### Histology and immunohistochemistry of PCLS

Tissue was fixed for up to 24 h at 4°C before samples were processed with an automated tissue processor (Tissue-Tek^®^ VIP^®^ 6, Sakura), embedded in paraffin, and cut in 3-µm sections. Hematoxylin and eosin (H&E) staining was performed according to standard protocols using Leica ST5020 Multistainer (Leica Biosystems GmbH, Nussloch, Germany).

Degenerating cells were detected using the TUNEL assay (DIG-11-dUTP, catalogue no. 11570013910, Roche; TdT-Enzyme, M1875, Promega). The assay was carried out on the automated Leica Bond RX™ platform (Leica Biosystems, Melbourne, Australia) and visualized using the Bond™ Polymer Refine Detection Kit [catalogue no. 37072; 3,3′ diaminobenzidine (DAB) as chromogen; Leica Biosystems, Newcastle, United Kingdom].

Images were created using the Axio Scan.Z1 slide scanner (×20 objective, Carl Zeiss Microscopy GmbH, Jena, Germany) and ZEN Blue software (version 3.7.5, Carl Zeiss Microscopy GmbH, Jena, Germany).

### Statistical analysis

GraphPad Prism 9.0 (GraphPad Software) was used to perform the statistical analyses. Statistical significance in the experimental data was calculated using one-way ANOVA with Benjamini–Krieger FDR correction, followed by paired Student’s *t*-test. *p* < 0.05 was considered significant. The levels of significance are indicated by *p < 0.05, **p < 0.01, *** *p* < 0.001, and **** *p* < <0.0001. Statistics are presented as mean ± SEM as indicated.

## Results

### TGF-β1 and TNF-α induce unique fibroblast activation

**Figure 1:**
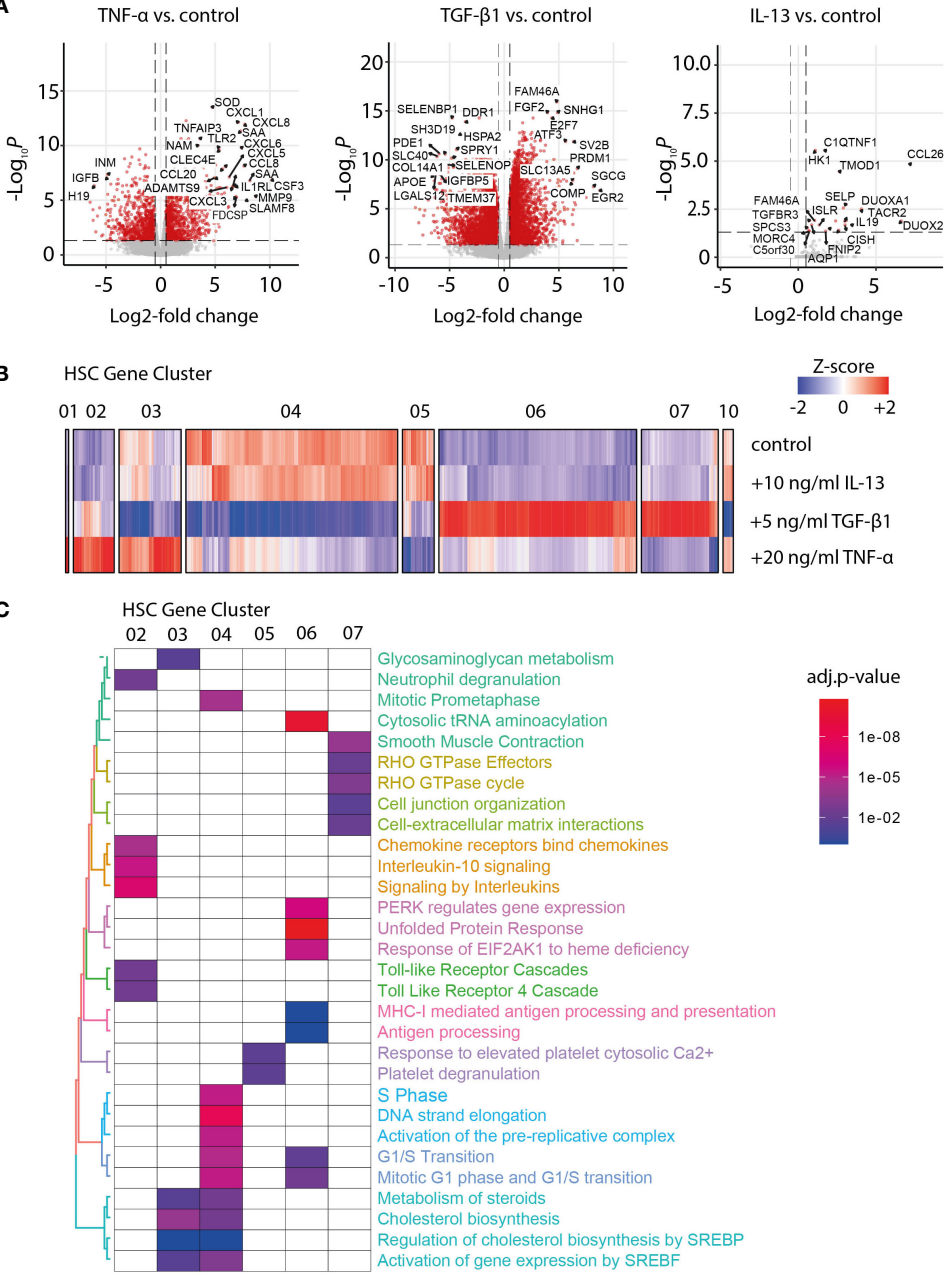
Transcriptome analysis of activated hepatic stellate cells (HSCs). **(A)** Volcano plot of differentially expressed genes in TNF-α-, TGF-β1-, and IL-13-activated HSC *versus* the untreated control. **(B)** Heat map of Z-score normalized gene expression after hierarchical clustering for gene clusters 01–07 and 10, as identified for control and IL-13-, TGF-β1-, and TNF-α-stimulated HSC. **(C)** Gene set enrichment analysis for the main gene clusters 01 to 07. The color code indicates the significance of the enrichment for the specified gene set in the corresponding cluster (adjusted *p*-value for multiple testing).

Liver fibrosis development is characterized by the transformation of HSC into myofibroblasts (MFB). To investigate the gradual increase of MFB activation during NASH, we performed a whole transcriptome analysis of primary human HSC treated with IL-13, TGFβ-1, or TNF-α for 24 h (**Figure 1A**). Next, we performed a gene cluster analysis to identify the co-regulated genes among the different activation conditions. Accordingly, 10 clusters of co-regulated genes were identified and functionally characterized by gene set enrichment analysis of the six main gene clusters (**Figures 1B, C**). As expected, TGF-β1 treatment induced a fibrotic response in HSC, indicated by upregulation of gene sets related to smooth muscle cell contraction and interaction with the extracellular matrix (**Figure 1C**). An overlap analysis with previously published human liver scRNA-Seq data (18) showed that the TGF-β1 response genes in HSC cluster 07 were expressed in the mesenchyme as well as the parenchymal cell types (**Supplementary Figure S1A–C**), underlining the partial specificity of the identified gene clusters for MFB. On the contrary, TNF-α treatment induced an inflammatory response in HSC, characterized by an overrepresentation of gene sets related to toll-like receptor, chemokine, and interleukin signaling, indicating the relevance of HSC in inflammation (**Figure 1C**). The IL-13 treatment induced only minor changes in gene expression compared to the control and did induce neither a fibrotic nor inflammatory response in HSC (**Figure 1A**).

### M-CSF differentiation induces a unique macrophage phenotype

**Figure 2:**
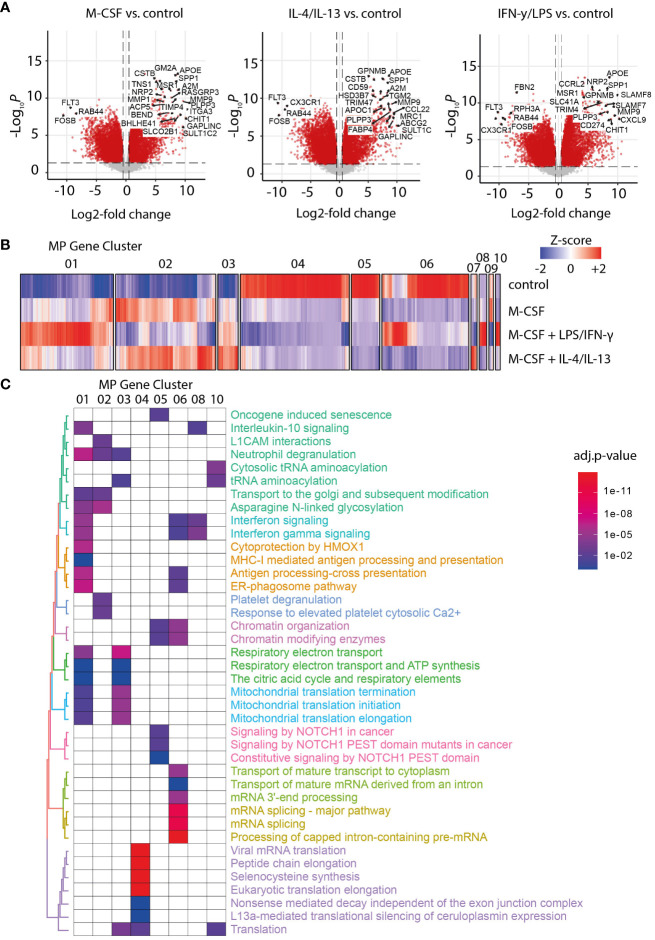
RNA sequencing analysis of macrophage activation patterns. **(A)** Volcano plot of differentially expressed genes in macrophage colony-stimulating factor 1 (M-CSF) and M-CSF with either IL-4/IL-13- or LPS/IFN-γ-activated MF *versus* untreated MoMF control. **(B)** Heat map of Z-score-normalized gene expression after hierarchical clustering for clusters 01 to 10. **(C)** Gene set enrichment analysis for the main gene clusters MF 01 to 07, 08, and 10. The color code indicates the significance of the enrichment for the specified gene set in the corresponding cluster (adjusted *p*-value for multiple testing).

Another characteristic of NASH is the expansion of MoMF in areas of inflammation in the liver. Transcriptomic signatures of monocytes and M-CSF-differentiated MoMF polarized with IFN-γ/LPS or IL-4/IL-13 were generated and subjected to hierarchical clustering. As expected, M-CSF treatment induced the upregulation of well-known macrophage markers such as SPP1, APOE, or GPNMB (**Figure 2A**) (28). Hierarchical clustering identified 10 clusters of co-regulated genes across the four different conditions (**Figure 2B**). Clusters 01, 06, and 08 contain interferon gamma-dependent genes as confirmed by gene set enrichment analysis (**Figure 2C**), while clusters 02 and 03 contain genes that are dependent on IL-4 and IL-13 polarization. Those genes of MoMF cluster 02 were found to be expressed by resident liver KC and mature TREM2^+^ MoMF according to previously published scRNA-Seq data from liver cirrhosis and controls (18). On the contrary, genes enriched in MoMF cluster 04, which are specific for unpolarized monocytes, were also highly expressed in CD14^+^ and CD16^+^ monocytes according to the human scRNA-Seq data (**Supplementary Figures 2A, B**). Other enriched gene sets indicate changes in citric acid cycle, ATP synthesis, or the translational state of macrophages (**Figure 2C**), which is in line with previous reports (29).

### NASH is characterized by gene signatures of myofibroblast and monocytes

**Figure 3:**
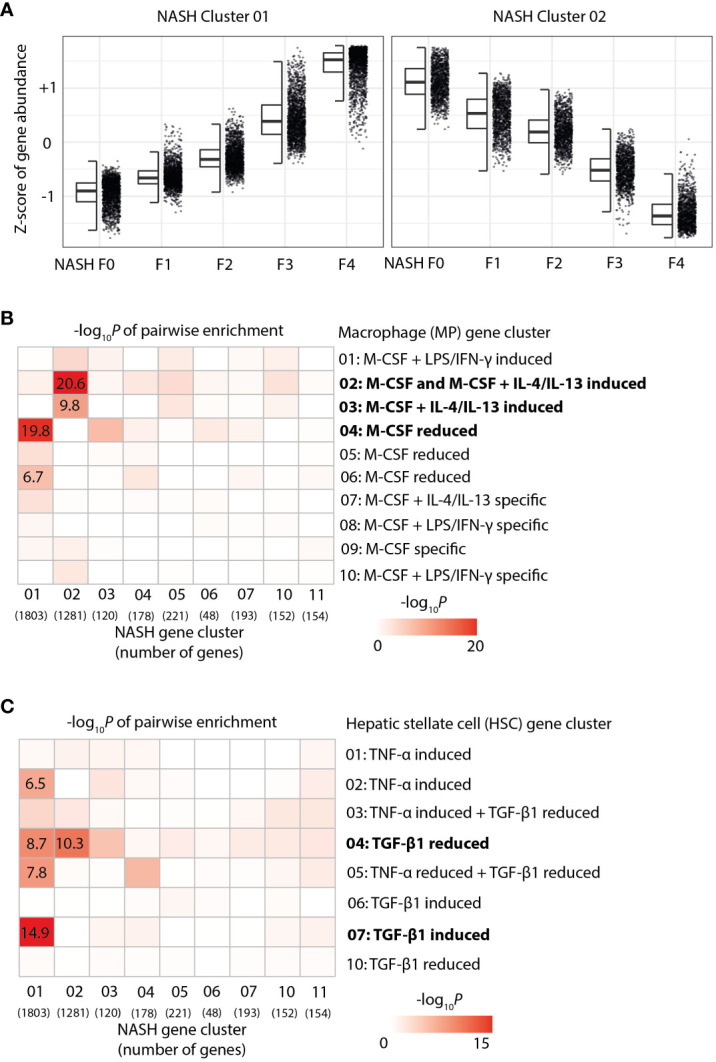
Enrichment of hepatic stellate cells (HSC) and macrophage gene signatures in human non-alcoholic steatohepatitis (NASH) liver biopsies. **(A)** Z-score-normalized gene expression by fibrosis stage F0 to F4 in the two main gene clusters—identified by hierarchical clustering from the RNA-Seq of human NASH liver biopsies. **(B)** Pairwise enrichment of NASH gene cluster with gene clusters from the activated macrophages. the color code indicates -log_10_ *p*-value. **(C)** Pairwise enrichment of NASH gene cluster with gene clusters from activated HSC. the color code indicates -log_10_ *p*-value of the enrichment score.

Furthermore, we systematically compared the identified gene signatures of *in vitro*-polarized HSC and MoMF with liver gene signatures from human NASH samples across the full spectrum of fibrosis from F0 to F4. Accordingly, we have re-analyzed the previously published RNA-Seq data (20) by hierarchical clustering, which ultimately led to the identification of two large clusters of genes that show a strong log–linear increase or decrease of expression with fibrosis stage (**Figure 3A**). Pairwise enrichment analysis of the fibrosis-dependent gene clusters with our MoMF-derived gene clusters revealed a significant loss of M-CSF + IL-4/IL-13 response genes (**Figure 3B**), indicating a continuous loss of this macrophage phenotype during NASH disease progression from F0 to F4. In contrast, the TGF-β1-dependent genes identified in our HSC activation assay were found to be strongly enriched in NASH cluster 01, which is increased with fibrosis (**Figure 3C**), reflecting the increasing population of TGF-β1-dependent MFB with disease worsening. Taken together, these data indicate that liver fibrosis progression, as a consequence of NASH, is characterized by loss of restorative M-CSF-dependent liver macrophages, in line with recent observations in NASH mouse models (30).

### Limited contribution of M-CSF-polarized macrophages to HSC activation

**Figure 4:**
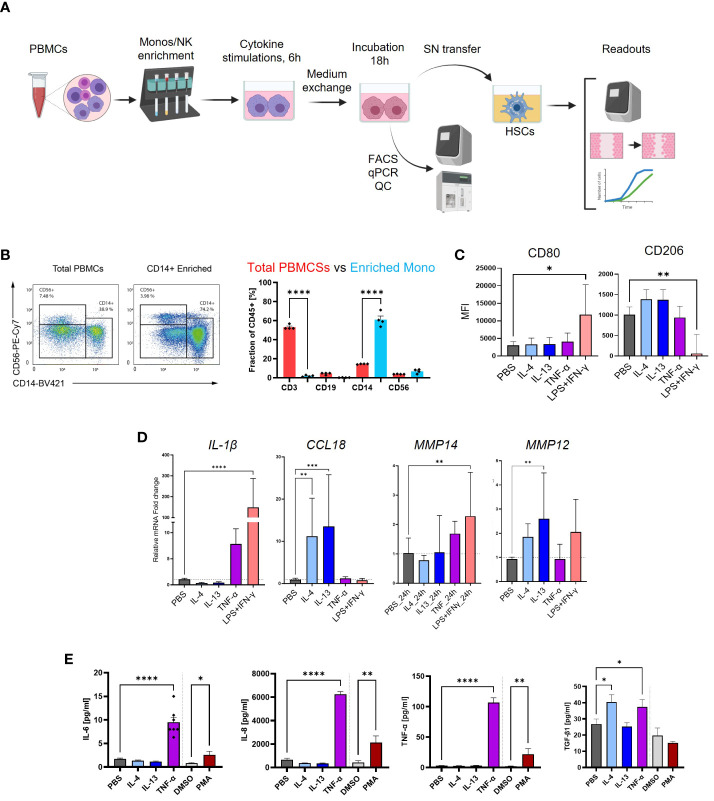
Experimental design and quality controls of monocyte-derived macrophages (MoMF) experiments. **(A)** Schematic illustration of the experimental workflow for monocytes, MoMF, and NK cells. **(B)** Enrichment of CD14^+^ monocytes by magnetic-activated cell sorting (MACS) determined by flow cytometry (FACS). Cell type proportions before (red) and after (blue) monocyte enrichment. **(C)** Cell surface markers, CD80 and CD260, measurement on M1- and M2-polarized MoMF. **(D)** qPCR quantification of cytokines/chemokines of M1–M2 MoMF. **(E)** macrophage colony-stimulating factor 1 (M-CSF)-differentiated macrophage-secreted IL-6, IL-8, TNF-α, and TGF-β1 as quantified by MSD-ELISA. One-way ANOVA with Benjamini–Krieger correction; *p < 0.05, **p < 0.01, ***p < 0.001, ****p < 0.0001.

**Figure 5:**
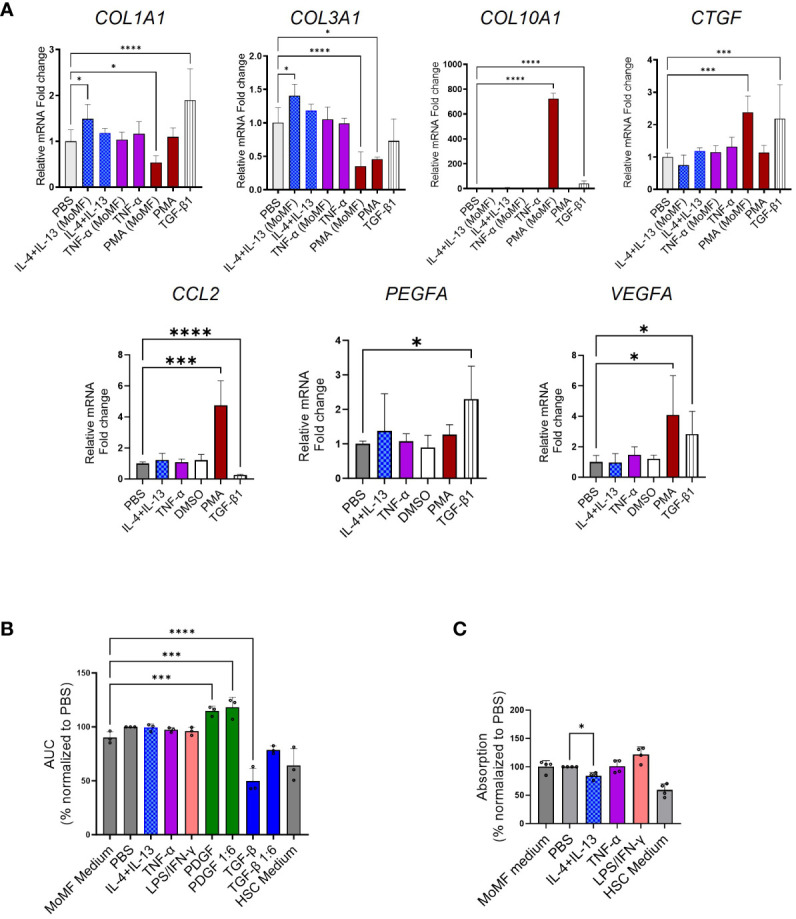
Limited fibrotic capacity of macrophage colony-stimulating factor 1 (M-CSF)-dependent macrophages. **(A)** Myofibroblast (MFB) activation after macrophage supernatant transfer as quantified by qPCR. **(B, C)** Bar plots presenting scratch assay and proliferation experiment. **(B)** The supernatant from stimulated monocyte-derived macrophages (MoMF) cells [from four independent peripheral blood mononuclear cells (PBMCs)] was used to activate hepatic stellate cells (HSCs) in the scratch assay. The area under the curve for all the stimulation conditions was calculated and used for the statistics. **(C)** The proliferation of HSCs following supernatant transfer assay (from three independent PBMCs) was qualified in the BrdU incorporation experiment in four independent HSCs. All data are displayed as mean ± SEM, *n* ≥ 8; data from three individual experiments. One-way ANOVA with Benjamini–Krieger correction; *p < 0.05, ***p < 0.001, ****p < 0.0001.

We aimed to investigate the potential of cell culture supernatant from differentiated and polarized macrophages in the activation of HSCs. To assess the fibrotic potential of MoMF, we isolated primary CD14+ monocytes from human PBMCs via MACS and differentiated for 7 days with 50 ng/mL recombinant human M-CSF, followed by 6 h of stimulation with either IL-4, IL-13, or TNF-α to induce unique polarization patterns, as depicted in **Figure 4A**. MoMF differentiation and polarization were confirmed by evaluating cell surface markers as well as the production and release of cytokines as IL-6, IL-8, TNF-α, and active TGF-β1 (**Figures 4B–E**). Furthermore, we confirmed macrophage differentiations by checking the mRNA expression of M1/M2-polarized secreted markers. The mRNA levels of IL-1β and MMP14, respectively, were significantly upregulated in LPS+IFN-γ-polarized MoMF, whereas higher mRNA levels of CCL18 and MMP12 were detected in IL-4/IL-13-treated MoMF (**Figure 4D**). In our experimental setting, in contrast to the classical inflammatory cytokines, the secretion of the anti-inflammatory and pro-fibrotic cytokine TGF-β1 was only moderately regulated following IL-4 or TNF-α stimulation (**Figure 4E**). The supernatant of the activated macrophages was then transferred to the primary human HSC. The gene expression analysis revealed that the supernatant from macrophages stimulated with TNF-α showed no induction of fibroblast activation; however, IL-4-IL-13 had a small effect on *COL1A1* and *COL3A1* expression (**Figure 5A**). The supernatant from PMA-hyperactivated MoMF induced a significant increase in *CTGF*, *CCL2*, *VEGFA*, and *COL10A1* expression and a significant decrease in *COL1A1* and *COL3A1* expression in HSC (**Figure 4A**). *COL10A1* is an extracellular matrix-related protein and has been associated with cancer-associated fibroblasts (31). These data demonstrate that, with our experimental setting, IL-4/IL-13-polarized MoMF lack the capacity to induce MFB activation of HSC and that the secretion of TGF-β1 alone is not robust enough to induce the pro-fibrotic capacity of macrophages.

To further evaluate the effect of supernatant from stimulated MoMF supernatant transfer on HSC activation, we performed scratch and proliferation assays after the supernatant transfer. No significant induction of HSC proliferation or migration, as markers of HSCs trans-differentiation, was observed (**Figures 5B, C**).

### Limited role of undifferentiated monocytes during HSC activation

**Figure 6:**
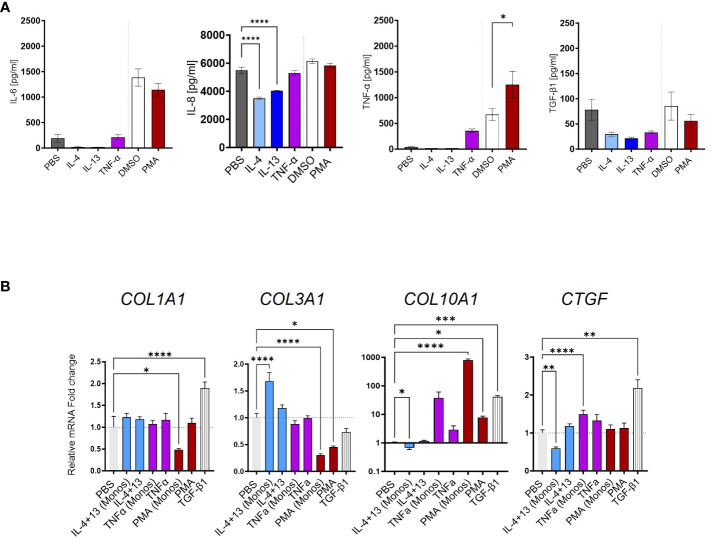
Limited fibrotic capacity of CD14+ monocytes. **(A)** IL-6, IL-8, TNF-α, and TGF-β1 secretion from CD14^+^ monocytes stimulated with IL-4, IL-13, TNF-α, or phorbol 12-myristate 13-acetate (PMA) as quantified by MSD-ELISA. **(B)** Myofibroblast (MFB) activation after monocyte supernatant transfer as measured by qPCR. All data are displayed as mean ± SEM, *n* ≥ 8; data from three individual experiments. One-way ANOVA with Benjamini–Krieger correction; *p < 0.05, **p < 0.01, ***p < 0.001, ****p < 0.0001.

Next, we investigated undifferentiated monocytes in a similar setup as for the MoMF. The cytokine measurement of IL-6, IL-8, and TNF-α in the supernatant showed limited inflammatory polarization of freshly isolated monocytes upon the different stimuli (**Figure 6A**). Accordingly, we found no correlation between TGF-β1 secretion and HSC trans-differentiation with the supernatant of IL-4-, IL-13-, or TNF-α-stimulated monocytes (**Figure 6B**). Only *COL3A1* gene expression was found to be increased by the supernatant of IL-4- and IL-13-stimulated monocytes, opposing the direction of TGF-β1 (**Figure 5B**). Differently from MoMF, the supernatant of TNF-α- or PMA-treated CD14^+^ monocytes did not induce a significant change of fibrotic response genes in HSC (**Figure 6B**). In summary, our data indicate a limited contribution of activated monocytes to pro-fibrotic HSC activation.

### Activated NK cells contribute to HSC activation

**Figure 7:**
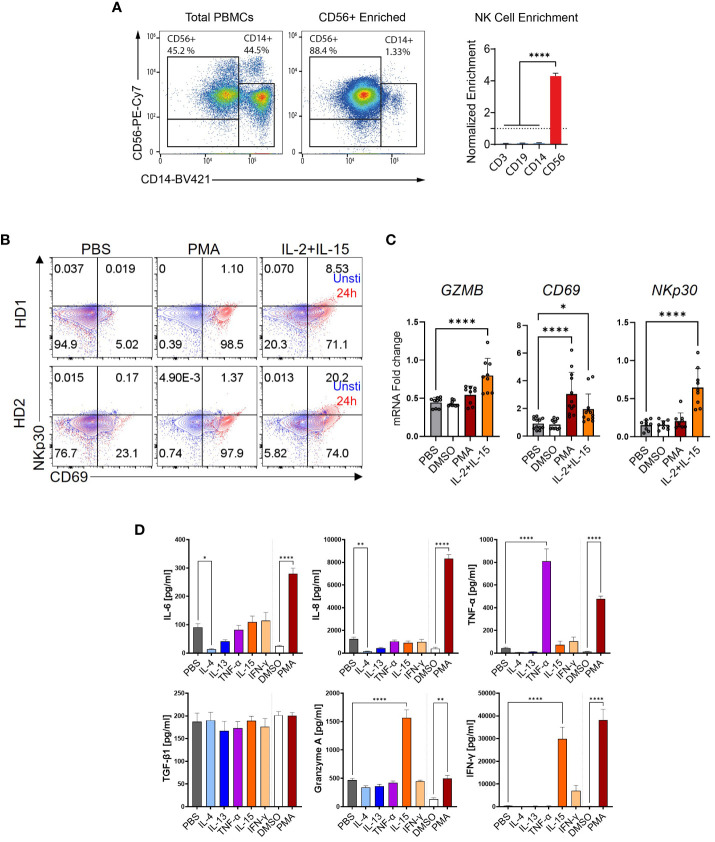
NK cell stimulation and activation. **(A)** Enrichment of CD56^+^ NK cells by magnetic-activated cell sorting (MACS) as quantified by flow cytometry (FACS). Normalized enrichment after MACS purification against the other cell type proportions before enrichment. **(B)** FACS plots of NK cell activation markers. The cell surface markers CD69 and NKp30 were stained and measured in NK cells stimulated with phorbol 12-myristate 13-acetate (PMA) or IL-2+IL-15. **(C)** Relative mRNA expression of NK cell activation markers. NK cells were stimulated for 6 h and used for the qPCR quantification of IL-1β, CD69, and NKp30. **(D)** CD56^+^ NK cells secreted IL-6, IL-8, TNF-α, TGF-β1, Granzyme A, and IFN-Υ as quantified by MSD-ELISA. One-way ANOVA with Benjamini–Krieger correction; *p < 0.05, **p < 0.01, ****p < 0.0001.

**Figure 8:**
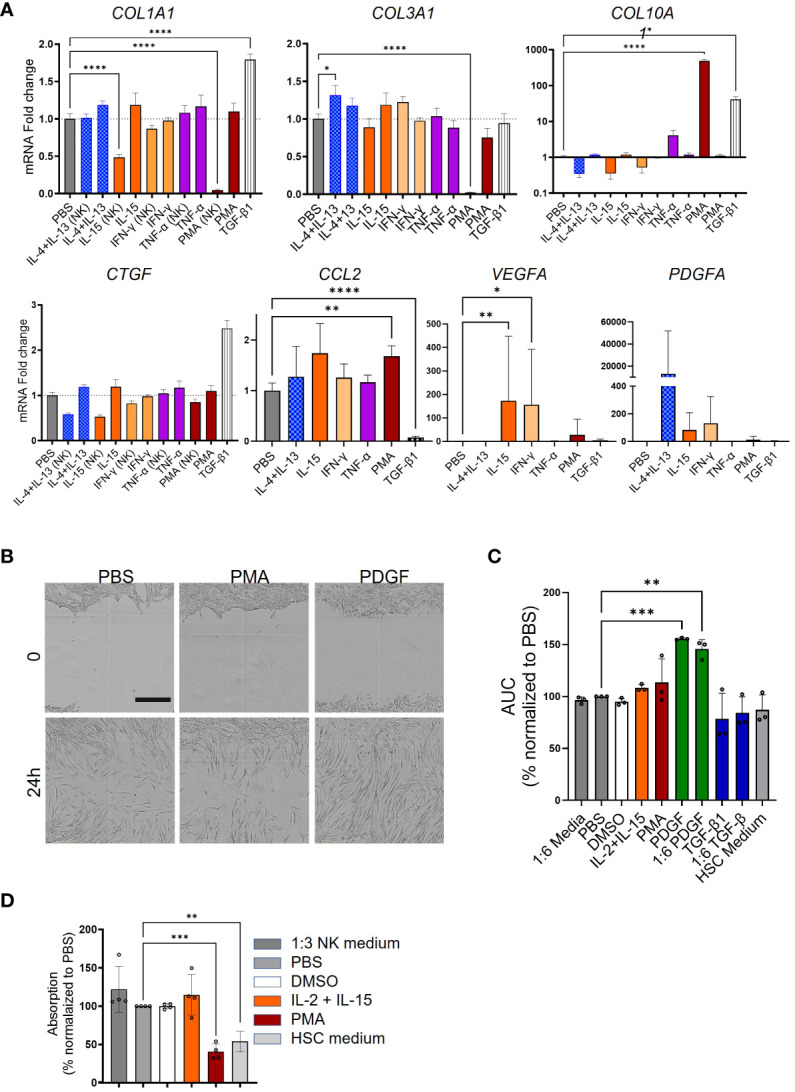
Variable response of NK cells to phorbol 12-myristate 13-acetate (PMA) hyperactivation. **(A)** Myofibroblast (MFB) activation after NK cell supernatant transfer as quantified by qPCR. *COL1A1*, *COL3A1*, *COL10A1*, *CCL2*, *PDGFA*, *VEGFA*, and *CTGF* were measured in NK cells stimulated with various stimuli. **(B)** Representative microscopy pictures of the scratch assay in response to phosphate-buffered saline (PBS), phorbol 12-myristate 13-acetate (PMA), or PDGF at two time points. Scale bar: 400 µm. **(C, D)** Bar plots presenting the scratch assay and the proliferation experiment. **(C)** Supernatant from stimulated NK cells [from four independent peripheral blood mononuclear cells (PBMCs)] was used to activate hepatic stellate cells (HSCs) in the scratch assay. The area under the curve (AUC) for all the stimulation conditions was calculated and used for the statistics. **(D)** The proliferation of HSCs following supernatant transfer assay (from three independent PBMCs) was qualified in the BrdU incorporation experiment in four independent HSCs. All data are displayed as mean ± SEM, *n* ≥ 8; data from three individual experiments. One-way ANOVA with Benjamini–Krieger correction; *p < 0.05, **p < 0.01, ***p < 0.001, ****p < 0.0001.

Next, we investigated the impact of NK cells on HSC activation. The experimental procedure is depicted in **Figure 4A**. CD56^+^ NK cells were enriched from PBMCs by MACS, their purity assessed by using FACS (**Figure 7A**), and stimulated with PMA, IL-15, or IFN-γ for 6 h. The medium was changed and the cells were incubated for another 16 h, and the supernatants were transferred to HSC. For quality control, the expression of some activation markers including CD69, NCR3 (natural cytotoxicity triggering receptor 3, also known as NKp30), and GZMB was analyzed by FACS and qPCR (**Figures 7B, C**). Furthermore, cytokine release by NK cells was quantified in NK cells following various stimulations As shown in **Figure 7D**, in contrast to the low secretion of TGF-β1 in MoMF and monocytes, the NK cells showed a higher background level of secreted TGF-β1, but no significant regulation upon release of stimuli. The supernatant of NK cells which were activated with IL-4, IL-13, and IL-15 reduced the HSC activation level compared to TGF-β1-stimulated cells (**Figure 8A**). Only PMA-hyperactivated NK cells induced a significant increase of *COL10A1* in HSCs (**Figure 8A**), like the observations in MoMF. Nevertheless, the PMA-stimulated NK cell supernatant led to a general decrease of pro-fibrotic activation in other marker genes measured (*COL1A1* and *COL3A1*) (**Figure 8A**). In line with the collagen mRNA expression data, we observed no significant induction of HSC proliferation and migration in the supernatant from stimulated NK cells (**Figures 8B–D**).

These data indicate that other immune cell populations besides MoMF could also modulate HSC to MFB differentiation during liver fibrosis progression.

### PMA induces HSC activation *ex vivo*

**Figure 9:**
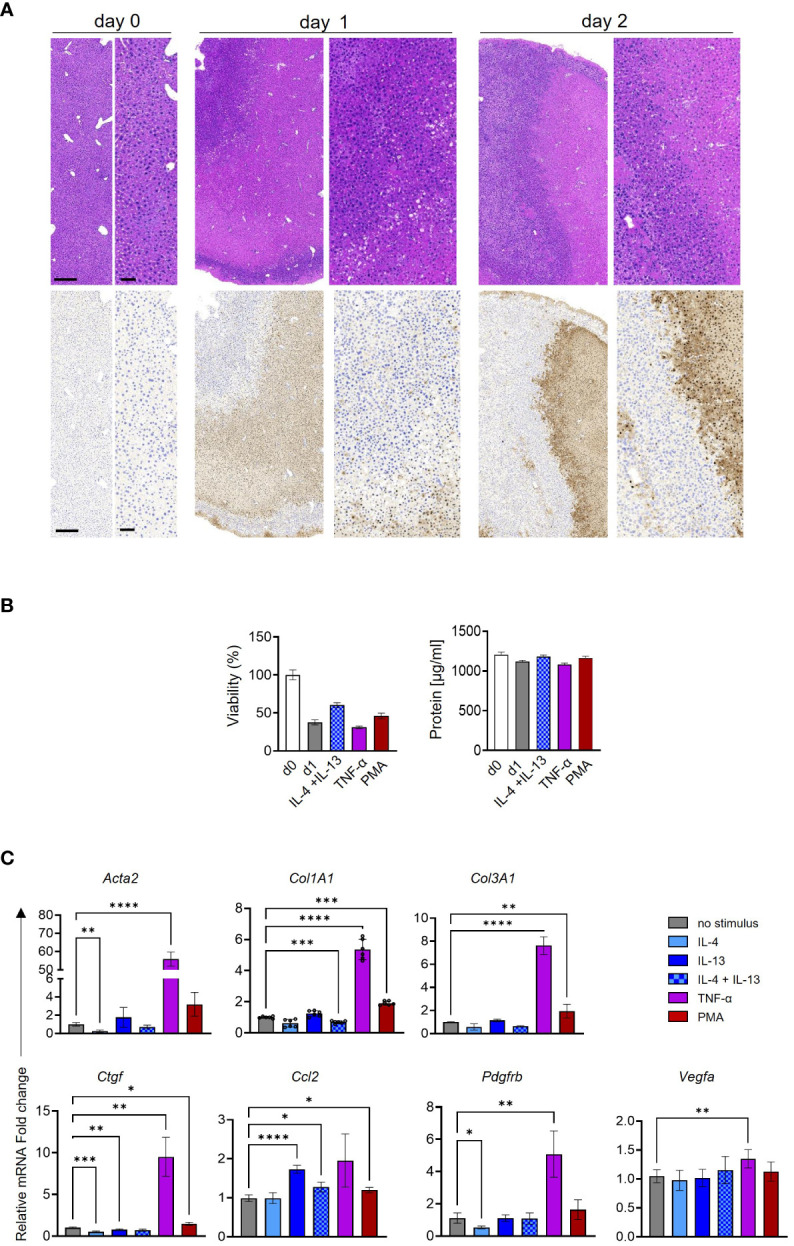
*Ex vivo* activation of murine hepatic stellate cells (HSCs) in precision-cut liver slices (PCLS). **(A)** Overview of tissue morphology in H&E (upper panel) and TUNEL expression (lower panel) in three independent PCLS. Bar scales: 250 µm in the left images and 100 µm in the right images. **(B)** Viability and total protein content of PCLS comparing day 0 (d0) to various treatments in day 1 (d1). **(C)** HSC activation markers were quantified following cytokine and phorbol 12-myristate 13-acetate (PMA) stimulation in three independent PCLS. One-way ANOVA with Benjamini–Krieger correction; *p < 0.05, **p < 0.01, ***p < 0.001, ****p < 0.0001.

Finally, to translate our *in vitro* findings to a more relevant *in vivo* condition, we assessed HSC activation in PCLS as a surrogate model. To prolong macrophage and monocyte viability, all PCLS were treated with M-CSF. The tissue morphology and viability of the PCLS samples were evaluated through H&E and TUNEL staining as well as measurement of total protein content and ATP release. H&E morphology was characterized by partial loss of cellular architecture with lysis of nuclear structures beginning on day 1; however, viable hepatic tissue was evident up to day 2 (**Figure 9A**). The morphological findings were confirmed by TUNEL staining distribution and extent. Moreover, no significant loss of total protein content and viability was pronounced between different treatments of PCLS at day 1 (**Figure 9B**). Various stimuli were added to each PCLS for 24 h, and HSC activation markers were quantified by qPCR (**Figure 9C**). The TNF-α treatment of PCLS induced a significant increase in almost all HSC activation markers. However, this observation might be due to the broad range of pro-fibrotic TNF-α effects observed in PCLS. In contrast to TNF-α, IL-4 + IL-13 stimulation only increased *Col1a1*, but not of other markers. The PMA-treated PCLS showed a significant increase in the mRNA expression of *Col1a1*, *Col3a1*, and *Ctgf* (**Figure 9C**), in line with our *in vitro* data.

Our *ex vivo* data confirmed a strong effect of PMA on activating HSCs, which could be due to the essential role of the PKC pathway in activating HSCs.

## Discussion

Our study aims to delineate the pro-fibrotic potential of the secretome of myeloid immune cells in the context of HSC activation and liver fibrosis progression. Macrophages are known for their high phenotypic plasticity and being involved in both acute and chronic liver diseases, including liver fibrosis (5, 32). It has been reported that macrophages promote fibrogenesis by secreting cytokines which consequently drive the trans-differentiation of HSCs (33, 34). Surprisingly, we found that, in our experimental conditions, IL-4/IL-13-stimulated MoMF failed to activate fibroblasts *in vitro* in contrast to PMA hyper-activated MoMF or NK cells. In addition, we did not observe an association between secreted TGF-β1 and fibroblast activation in supernatant transfer assays. This indicates that, in the presence of other soluble mediators, TGF-β1 could be less relevant than if used alone (35). These findings are of particular interest as previous studies have described scar-associated macrophages in human cirrhotic liver, with a high capacity to induce HSC activation *in vitro* (18). The comparison of RNA-Seq-derived gene signatures indicates that NASH progression is characterized by a lack of restorative IL-4/IL-13-stimulated MoMF. In line with that, Stutchfield et al. showed that restorative M-CSF-differentiated monocytes and macrophages could provide therapeutic opportunities to improve recovery from acute liver injury (36). In addition, MoMF are described to play a key role in liver fibrosis reversal (37). Moreover, previous studies also suggested that liver cirrhosis could be treated by increasing the amount of liver macrophages via recombinant M-CSF treatment (10). Although our data indicate no direct fibrotic potential of M-CSF-polarized MoMF, which is of great interest in light of the recent discussions regarding the heterogenous role of MoMF in liver fibrosis (28). A recent study disclosed a pro-fibrogenic role for MoMF population expressing CCR2 (38).

Liver macrophages have often been described as the main drivers of HSC activation and thus as key players in liver fibrosis progression. This correlation has been linked to reduced inflammation and fibrosis in the absence of infiltrating monocytes in *Ccr2^-/-^* mice or by using inhibitors of CCR2 or the ligand CCL2 (39–41). However, this concept has failed to translate into a beneficial clinical outcome (42). In line with our findings, the effect of various immune cells in activating HSCs in co-culture models has been studied by Zimmermann and Pastore (43, 44).

Additional functional studies might be needed to better understand the limited fibrotic potential of MoMF compared to that of tissue-resident KC or the recently described LAM (15, 45). One limitation prior to such investigations is the current lack of differentiation protocols for KC- or LAM-like macrophages *in vitro*. Future studies could also focus on identifying the key drivers of HSC activation, aside from TGF-β1, by in-depth secretome analysis. Taken together, our data highlight that TGF-β1 expression or secretion does not necessarily indicate a pro-fibrotic role of macrophages and that future studies will be needed to further dissect the interplay between myeloid immune cells and HSC activation in liver fibrosis.

## Data availability statement

The RNASeq data sets used in this study can be found in the Gene Expression Omnibus under the accession numbers GSE135251 for the previously published human NASH dataset (20) and GSE228088 for the HSC and MoMF assay data sets from this study. The status of the RNASeq data record has been changed to publicly available through the following link: https://eur03.safelinks.protection.outlook.com/?url=https%3A%2F%2Fwww.ncbi.nlm.nih.gov%2Fgeo%2Fquery%2Facc.cgi%3Facc%3DGSE228088&data=05%7C02%7Cehsan.bahrami%40boehringer-ingelheim.com%7C7aa93588d4e0433aa06308dc499be732%7Ce1f8af86ee954718bd0d375b37366c83%7C0%7C0%7C638466181144385704%7CUnknown%7CTWFpbGZsb3d8eyJWIjoiMC4wLjAwMDAiLCJQIjoiV2luMzIiLCJBTiI6Ik1haWwiLCJXVCI6Mn0%3D%7C0%7C%7C%7C&sdata=7XdRAmHtm73SfLDyvPpWsL8JMUjZec6kayyDg3L9pJw%3D&reserved=0;https://www.ncbi.nlm.nih.gov/geo/query/acc.cgi?acc=GSE228088.

## Ethics statement

The studies involving humans were approved by Boehringer-Ingelheim Research and development ethics committee. The studies were conducted in accordance with the local legislation and institutional requirements. Written informed consent for participation in this study was provided by the participants’ legal guardians/next of kin. Animal experiments were conducted in compliance with the German and European Animal Welfare Acts and approved by the local German authority.

## Author contributions

JS: designed and performed the *in vitro* experiments and wrote the manuscript; AS-K: analysis of RNA-Seq data and wrote the manuscript; SG: performed experiments on PCLS and analyzed data; MM: analysis of RNA-Seq data and method description; JR: designed the *in vitro* RNA-Seq experiments; SB and DB: assisted in RNA extraction and qPCR experiments; TS and CL: Performed H&E and TUNEL staining and evaluation on the PCLS; VF: designed, and supervised and analyzed experiments on PCLS; ES: design and supervision of RNA-Seq analysis and wrote the manuscript; OK: guided and designed the experiments and wrote the manuscript; EB: designed and analyzed experiments, wrote and revised the manuscript. All authors contributed to the article and approved the submitted version.

## References

[1] Parthasarathy G Revelo X Malhi H. Pathogenesis of nonalcoholic steatohepatitis: an overview. Hepatol Commun. (2020) 4:478–92. doi: 10.1002/hep4.1479 PMC7109346 32258944

[2] Kisseleva T Brenner D. Molecular and cellular mechanisms of liver fibrosis and its regression. Nat Rev Gastroentero. (2021) 18:151–66. doi: 10.1038/s41575-020-00372-7 33128017

[3] Guillot A Tacke F. Liver macrophages: old dogmas and new insights. Hepatol Commun. (2019) 3:730–43. doi: 10.1002/hep4.1356 PMC6545867 31168508

[4] Huby T Gautier EL. Immune cell-mediated features of non-alcoholic steatohepatitis. Nat Rev Immunol. (2021) 22:429–43. doi: 10.1038/s41577-021-00639-3 34741169 PMC8570243

[5] Schwabe RF Tabas I Pajvani UB. Mechanisms of fibrosis development in NASH. Gastroenterology. (2020) 158:1913–28. doi: 10.1053/j.gastro.2019.11.311 PMC7682538 32044315

[6] Hao C Xie Y Peng M Ma L Zhou Y Zhang Y. Inhibition of connective tissue growth factor suppresses hepatic stellate cell activation in *vitro* and prevents liver fibrosis in *vivo* . Clin Exp Med. (2014) 14:141–50. doi: 10.1007/s10238-013-0229-6 23456538

[7] Huang G Brigstock DR. Regulation of hepatic stellate cells by connective tissue growth factor. Front Biosci. (2012) 17:2495. doi: 10.2741/4067 22652794

[8] Ma P-F Gao C-C Yi J Zhao J-L Liang S-Q Zhao Y. Cytotherapy with M1-polarized macrophages ameliorates liver fibrosis by modulating immune microenvironment in mice. J Hepatol. (2017) 67:770–9. doi: 10.1016/j.jhep.2017.05.022 28596109

[9] Pouyanfard S Meshgin N Cruz LS Diggle K Hashemi H Pham TV. Human induced pluripotent stem cell-derived macrophages ameliorate liver fibrosis. Stem Cells. (2021) 39:1701–17. doi: 10.1002/stem.3449 34460131

[10] Moroni F Dwyer BJ Graham C Pass C Bailey L Ritchie L. Safety profile of autologous macrophage therapy for liver cirrhosis. Nat Med. (2019) 25:1560–5. doi: 10.1038/s41591-019-0599-8 31591593

[11] van de Laar L Saelens W De Prijck S Martens L Scott CL Van Isterdael G. Yolk sac macrophages, fetal liver, and adult monocytes can colonize an empty niche and develop into functional tissue-resident macrophages. Immunity. (2016) 44:755–68. doi: 10.1016/j.immuni.2016.02.017 26992565

[12] Triantafyllou E Woollard KJ McPhail MJW Antoniades CG Possamai LA. The role of monocytes and macrophages in acute and acute-on-chronic liver failure. Front Immunol. (2018) 9:2948. doi: 10.3389/fimmu.2018.02948 30619308 PMC6302023

[13] Li T Yang Y Song H Li H Cui A Liu Y. Activated NK cells kill hepatic stellate cells via p38/PI3K signaling in a TRAIL-involved degranulation manner. J Leukoc Biol. (2019) 105:695–704. doi: 10.1002/jlb.2a0118-031rr 30748035

[14] Zhang Y Wu Y Shen W Wang B Yuan X. Crosstalk between NK cells and hepatic stellate cells in liver fibrosis. Mol Med Rep. (2022) 25:208. doi: 10.3892/mmr.2022.12724 35506449 PMC9133963

[15] Guilliams M Bonnardel J Haest B Vanderborght B Wagner C Remmerie A. Spatial proteogenomics reveals distinct and evolutionarily conserved hepatic macrophage niches. Cell. (2022) 185:379–396.e38. doi: 10.1016/j.cell.2021.12.018 35021063 PMC8809252

[16] Hendrikx T Porsch F Kiss MG Rajcic D Papac-Miličević N Hoebinger C. Soluble TREM2 levels reflect the recruitment and expansion of TREM2+ macrophages that localize to fibrotic areas and limit NASH. J Hepatol. (2022) 77(5):1373–85. doi: 10.1016/j.jhep.2022.06.004 35750138

[17] Scott CL Zheng F Baetselier PD Martens L Saeys Y Prijck SD. Bone marrow-derived monocytes give rise to self-renewing and fully differentiated Kupffer cells. Nat Commun. (2016) 7:10321. doi: 10.1038/ncomms10321 26813785 PMC4737801

[18] Ramachandran P Dobie R Wilson-Kanamori JR Dora EF Henderson BEP Luu NT. Resolving the fibrotic niche of human liver cirrhosis at single-cell level. Nature. (2019) 575:512–8. doi: 10.1038/s41586-019-1631-3 PMC6876711 31597160

[19] Moore JK Mackinnon AC Wojtacha D Pope C Fraser AR Burgoyne P. Phenotypic and functional characterization of macrophages with therapeutic potential generated from human cirrhotic monocytes in a cohort study. Cytotherapy. (2015) 17:1604–16. doi: 10.1016/j.jcyt.2015.07.016 PMC4596388 26342993

[20] Govaere O Cockell S Tiniakos D Queen R Younes R Vacca M. Transcriptomic profiling across the nonalcoholic fatty liver disease spectrum reveals gene signatures for steatohepatitis and fibrosis. Sci Transl Med. (2020) 12:eaba4448. doi: 10.1126/scitranslmed.aba4448 33268509

[21] Schlager S Salomon C Olt S Albrecht C Ebert A Bergner O. Inducible knock-out of BCL6 in lymphoma cells results in tumor stasis. Oncotarget. (2020) 11:875–90. doi: 10.18632/oncotarget.27506 PMC7061739 32180900

[22] Pantano L Agyapong G Shen Y Zhuo Z Fernandez-Albert F Rust W. Molecular characterization and cell type composition deconvolution of fibrosis in NAFLD. Sci Rep-uk. (2021) 11:18045. doi: 10.1038/s41598-021-96966-5 PMC8433177 34508113

[23] Blighe K Rana S Lewis M. EnhancedVolcano: Publication-ready volcano Plots with Enhanced Colouring and Labeling . R package version 1.12.0. Available online at: https://github.com/kevinblighe/EnhancedVolcano (Accessed 2021).

[24] Gu Z Eils R Schlesner M. Complex heatmaps reveal patterns and correlations in multidimensional genomic data. Bioinformatics. (2016) 32:2847–9. doi: 10.1093/bioinformatics/btw313 27207943

[25] Gillespie M Jassal B Stephan R Milacic M Rothfels K Senff-Ribeiro A. The reactome pathway knowledgebase 2022. Nucleic Acids Res. (2021) 50:gkab1028–. doi: 10.1093/nar/gkab1028 PMC8689983 34788843

[26] Yu G Wang L-G Han Y He Q-Y. clusterProfiler: an R package for comparing biological themes among gene clusters. OMICS. (2012) 16:284–7. doi: 10.1089/omi.2011.0118 PMC3339379 22455463

[27] Yu G. Enrichplot: Visualization of Functional Enrichment Result . R package version 1.14.1. Available online at: https://yulab-smu.top/biomedical-knowledge-mining-book/ (Accessed 2022).

[28] Guilliams M Scott CL. Liver macrophages in health and disease. Immunity. (2022) 55:1515–29. doi: 10.1016/j.immuni.2022.08.002 36103850

[29] Xue J Schmidt SV Sander J Draffehn A Krebs W Quester I. Transcriptome-based network analysis reveals a spectrum model of human macrophage activation. Immunity. (2014) 40:274–88. doi: 10.1016/j.immuni.2014.01.006 PMC3991396 24530056

[30] Wang X He Q Zhou C Xu Y Liu D Fujiwara N. Prolonged hypernutrition impairs TREM2-dependent efferocytosis to license chronic liver inflammation and NASH development. Immunity. (2022). doi: 10.1016/j.immuni.2022.11.013 PMC9839616 36521495

[31] Andriani F Landoni E Mensah M Facchinetti F Miceli R Tagliabue E. Diagnostic role of circulating extracellular matrix-related proteins in non-small cell lung cancer. BMC Cancer. (2018) 18:899. doi: 10.1186/s12885-018-4772-0 30227835 PMC6145327

[32] Heymann F Tacke F. Immunology in the liver — from homeostasis to disease. Nat Rev Gastroentero. (2016) 13:88–110. doi: 10.1038/nrgastro.2015.200 26758786

[33] Li H Zheng H-W Chen H Xing Z-Z You H Cong M. Hepatitis B virus particles preferably induce Kupffer cells to produce TGF-β1 over pro-inflammatory cytokines. Digest Liver Dis. (2012) 44:328–33. doi: 10.1016/j.dld.2011.11.005 22177317

[34] Pradere J Kluwe J Minicis S Jiao J Gwak G Dapito DH. Hepatic macrophages but not dendritic cells contribute to liver fibrosis by promoting the survival of activated hepatic stellate cells in mice. Hepatology. (2013) 58:1461–73. doi: 10.1002/hep.26429 PMC3848418 23553591

[35] Frangogiannis NG. Transforming growth factor–β in tissue fibrosis. J Exp Med. (2020) 217:e20190103. doi: 10.1084/jem.20190103 32997468 PMC7062524

[36] Stutchfield BM Antoine DJ Mackinnon AC Gow DJ Bain CC Hawley CA. CSF1 restores innate immunity after liver injury in mice and serum levels indicate outcomes of patients with acute liver failure. Gastroenterology. (2015) 149:1896–1909.e14. doi: 10.1053/j.gastro.2015.08.053 26344055 PMC4672154

[37] Ramachandran P Pellicoro A Vernon MA Boulter L Aucott RL Ali A. Differential Ly-6C expression identifies the recruited macrophage phenotype, which orchestrates the regression of murine liver fibrosis. Proc Natl Acad Sci. (2012) 109:E3186–95. doi: 10.1073/pnas.1119964109 PMC3503234 23100531

[38] Bartneck M Koppe C Fech V Warzecha KT Kohlhepp M Huss S. Roles of CCR2 and CCR5 for hepatic macrophage polarization in mice with liver parenchymal cell-specific NEMO deletion. Cell Mol Gastroenterol Hepatol. (2021) 11:327–47. doi: 10.1016/j.jcmgh.2020.08.012 PMC7779787 32896623

[39] Karlmark KR Weiskirchen R Zimmermann HW Gassler N Ginhoux F Weber C. Hepatic recruitment of the inflammatory Gr1+ monocyte subset upon liver injury promotes hepatic fibrosis. Hepatology. (2009) 50:261–74. doi: 10.1002/hep.22950 19554540

[40] Baeck C Wehr A Karlmark KR Heymann F Vucur M Gassler N. Pharmacological inhibition of the chemokine CCL2 (MCP-1) diminishes liver macrophage infiltration and steatohepatitis in chronic hepatic injury. Gut. (2012) 61:416. doi: 10.1136/gutjnl-2011-300304 21813474

[41] Ehling J Bartneck M Wei X Gremse F Fech V Möckel D. CCL2-dependent infiltrating macrophages promote angiogenesis in progressive liver fibrosis. Gut. (2014) 63:1960. doi: 10.1136/gutjnl-2013-306294 24561613 PMC4216733

[42] Vuppalanchi R Noureddin M Alkhouri N Sanyal AJ. Therapeutic pipeline in nonalcoholic steatohepatitis. Nat Rev Gastroentero. (2021) 18:373–92. doi: 10.1038/s41575-020-00408-y 33568794

[43] Zimmermann HW Seidler S Nattermann J Gassler N Hellerbrand C Zernecke A. Functional contribution of elevated circulating and hepatic non-classical CD14+CD16+ Monocytes to inflammation and human liver fibrosis. PloS One. (2010) 5:e11049. doi: 10.1371/journal.pone.0011049 20548789 PMC2883575

[44] Pastore M Caligiuri A Raggi C Navari N Piombanti B Maira GD. Macrophage MerTK promotes profibrogenic cross-talk with hepatic stellate cells via soluble mediators. JHEP Rep. (2022) 4:100444. doi: 10.1016/j.jhepr.2022.100444 35252828 PMC8891698

[45] Jaitin DA Adlung L Thaiss CA Weiner A Li B Descamps H. Lipid-associated macrophages control metabolic homeostasis in a trem2-dependent manner. Cell. (2019) 178:686–698.e14. doi: 10.1016/j.cell.2019.05.054 31257031 PMC7068689

