# Supplementary material for: Diverse potential of secretome from natural killer cells and monocyte-derived macrophages in activating stellate cells
Source: Front Immunol. 2024 Apr 3;15:1232070. doi: 10.3389/fimmu.2024.1232070 (PMC11025356; doi:10.3389/fimmu.2024.1232070)
Supplement: Supplementary file 1 [file DataSheet_1.pdf]

Supplemental Figure 1

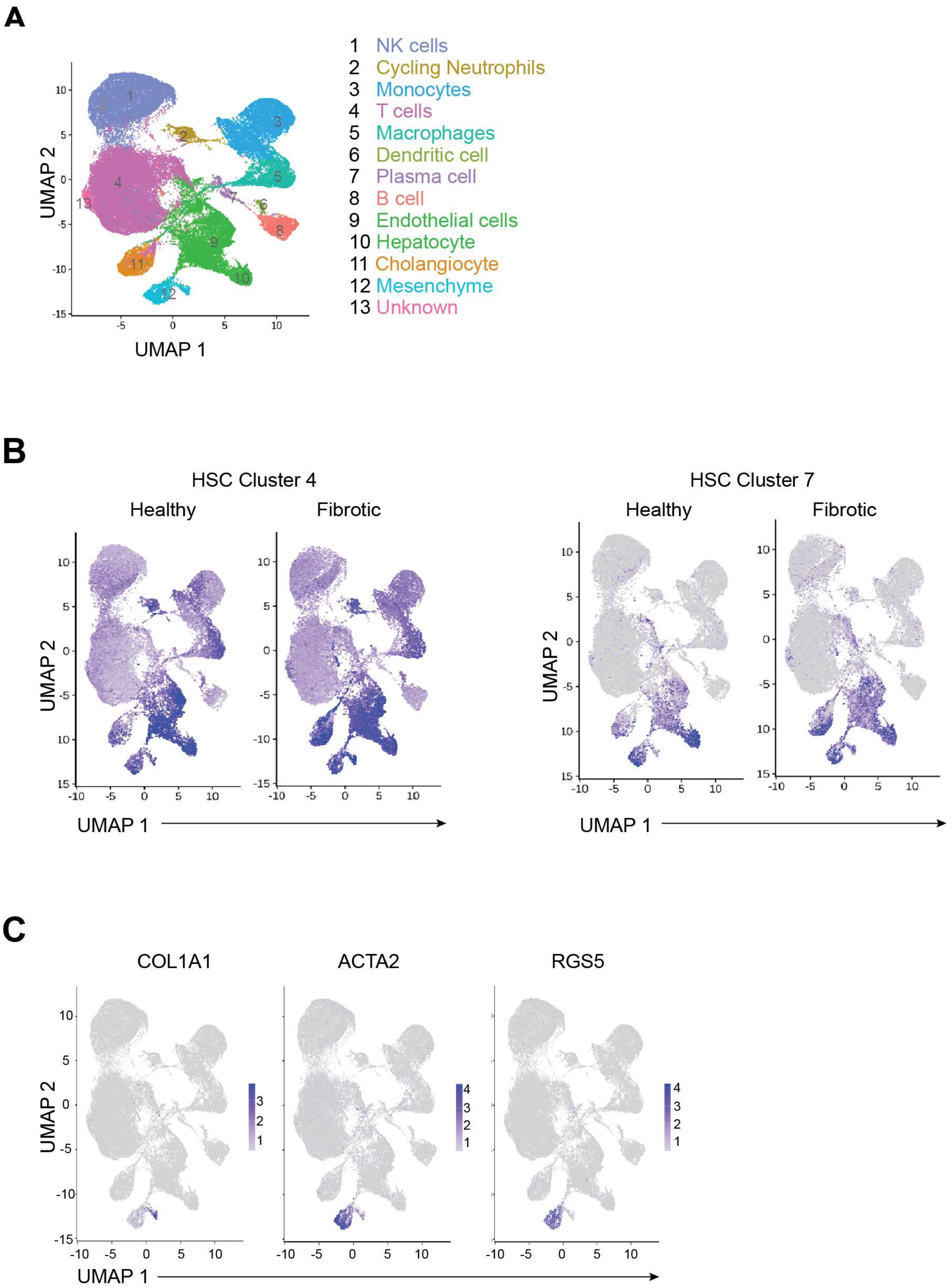

Supplementary Figure 1. Overlap of hepatic stellate cell clusters with human immune-enriched liver scRNASeq data. (A) Umap plot showing all 13 identified liver cell types. (B) Aggregated gene expression scores of HSC cluster 04 and 07. (C) UMAP plots showing the expression of three HSC marker genes *COL1A1, ACTA2* and *RGS5*

Supplemental Figure 2

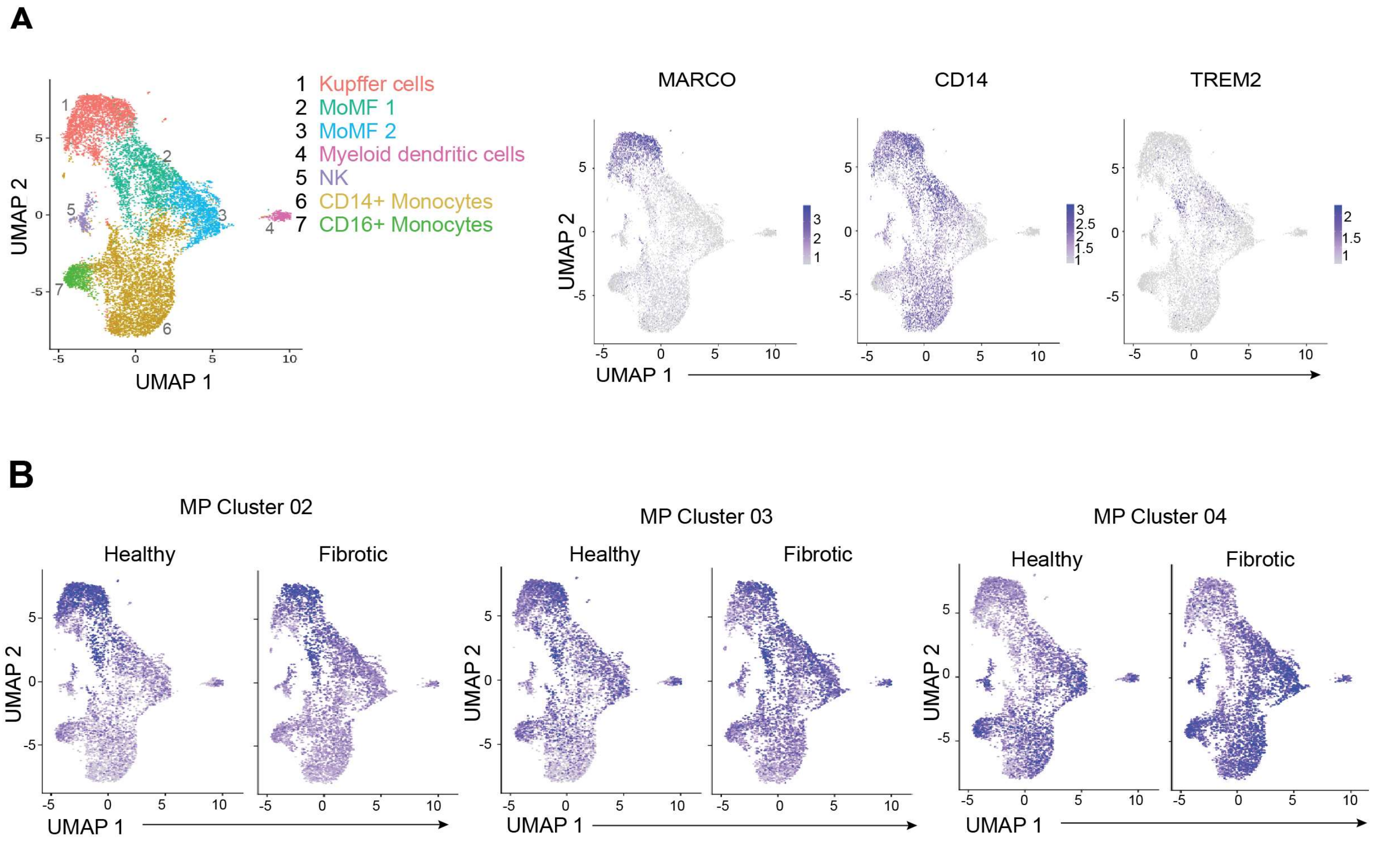

Supplementary Figure 2. Overlap of macrophage clusters with scRNASeq data. (A) Umap plot showing 7 identified monocyte and macrophage clusters and the expression of the three corresponding marker genes *MARCO*, *CD14* and *TREM2*. (B) Aggregated gene expression scores of the genes in the three macrophage clusters 02, 03 and 04 projected onto the monocyte and macrophage scRNASeq data subset.
